# Role of microRNAs in Congenital Diaphragmatic Hernia-Associated Pulmonary Hypertension

**DOI:** 10.3390/ijms24076656

**Published:** 2023-04-03

**Authors:** Flaminia Pugnaloni, Irma Capolupo, Neil Patel, Paola Giliberti, Andrea Dotta, Pietro Bagolan, Florian Kipfmueller

**Affiliations:** 1 Neonatal Intensive Care Unit, Bambino Gesù Children Hospital, Instituti di Ricovero e Cura a Carattere Scietifico (IRCCS), 00165 Rome, Italy; 2 Department of Neonatology, The Royal Hospital for Children, Glasgow G51 4TF, UK; 3 Area of Fetal, Neonatal and Cardiological Sciences Children’s Hospital Bambino Gesù-Research Institute, 00165 Rome, Italy; 4 Department of Systems Medicine, University of Rome “Tor Vergata”, 00165 Rome, Italy; 5 Department of Neonatology and Pediatric Intensive Care, Children’s Hospital, University of Bonn, 53127 Bonn, Germany

**Keywords:** epigenetics, microRNAs, pulmonary hypertension, congenital diaphragmatic hernia

## Abstract

Epigenetic regulators such as microRNAs (miRNAs) have a key role in modulating several gene expression pathways and have a role both in lung development and function. One of the main pathogenetic determinants in patients with congenital diaphragmatic hernia (CDH) is pulmonary hypertension (PH), which is directly related to smaller lung size and pulmonary microarchitecture alterations. The aim of this review is to highlight the importance of miRNAs in CDH-related PH and to summarize the results covering this topic in animal and human CDH studies. The focus on epigenetic modulators of CDH-PH offers the opportunity to develop innovative diagnostic tools and novel treatment modalities, and provides a great potential to increase researchers’ understanding of the pathophysiology of CDH.

## 1. Introduction

Congenital diaphragmatic hernia (CDH) is a complex congenital anomaly with a global prevalence of ~2.3 per 10,000 live births [1]; despite advances in global management of fetuses and newborns with CDH, it is still associated with significant morbidity and mortality [2,3]. Two of the main determinants of CDH-related mortality and morbidity are pulmonary hypoplasia and hypertension (PH) [4] which are closely correlated to prenatal lung size. Smaller lung size and pulmonary microarchitecture alterations characterize the abnormal lung development in CDH. Lung histologic irregularities result in functional alteration including compromised vasoreactivity [5,6]. The genetic background of CDH appears to be highly variable and still largely unknown, but several studies have focused on the potential role of epigenetic modifiers on the severity and the various phenotypes of CDH.

MicroRNAs (miRNAs) are small, noncoding RNAs (approximately 22 nucleotides) that are master regulators of the human genome, acting at the post-transcription level. MiRNAs are essential for normal organogenesis and show very complex time- and tissue-dependent expression patterns. The role of miRNAs in CDH-related PH is still poorly studied.

PH is defined as sustained supranormal pulmonary arterial pressure that leads to an altered pulmonary circulation with subsequent abnormal gas exchange and a ventilation–perfusion mismatch of variable severity and it has been demonstrated as a crucial predictor of adverse outcome [7,8,9,10].

Fetoscopic endoluminal tracheal occlusion (FETO) has been developed as a prenatal strategy in CDH, offered to women carrying singleton fetuses with isolated left or right CDH with the aim of accelerate lung growth by creating stretch within the lung parenchyma [11].

Recently published randomized controlled trials (NIH NCT01240057) in fetuses with left-sided CDH observed a significant increase in survival to discharge for those fetuses with severe CDH treated with FETO [12]. In contrast, the trial involving fetuses with moderate left-sided CDH failed to demonstrate a significant benefit of this procedure [13].

Conversely, FETO increased the risks of premature rupture of membranes and preterm birth, which laid the foundation for the search of less invasive solutions for prenatal treatment of pulmonary hypoplasia and early management of PH in CDH.

To date, more than 1800 miRNAs have been identified in human beings and aberrant miRNA expression has been associated with several commonly encountered diseases including diabetes, cancer and cardiovascular disease [14].

miRNAs demonstrate a regulatory involvement in several aspects of lung development and function acting both at a cellular and extracellular level. Extracellular miRNAs can freely circulate in the blood stream, be protein-bound or be vehiculated in extracellular vesicles (EVs). EVs play pivotal role in gene expression regulation. 

Research into the functional relevance of miRNAs on lung biology (lung development and function) and diseases is growing with both the diagnostic potential of altered miRNA expression levels and the therapeutic potential of miRNAs as areas of interest.

The aim of this review is to summarize the current evidence for the role of microRNAs in both animal models and humans with CDH and CDH-related pulmonary disease. A summary of relevant investigations is provided for the reader in Table 1 and Table 2. Considering these studies to date, we then critically discuss the future potential of miRNAs in CDH and priorities for future research. 

## 2. Role of miRNAs in CDH Related Pulmonary Hypertension

### 2.1. Animal CDH Models

Investigation in animal models have demonstrated that miRNAs are involved in several stages of lung development, and that altered miRNA expression can lead to postnatal diseases.

Lung development is a complex biological process characterized by five consecutive developmental stages: The two early stages (embryonic and pseudo-glandular) are fundamental for lung branching morphogenesis whereas the last three (canalicular, terminal saccular and alveolar) are characterized by progressive vascularization and air–blood interface formation.

The nitrofen-induced rat model has been used in recent decades to simulate the anomalies of CDH [24]. When nitrofen (2,4-dichlorophenyl- p -nitrophenyl ether) is administered to pregnant rats on the 9th day of gestation (before the lungs and diaphragm are formed) 70% of fetuses will develop CDH and 100% of them will develop PH. Several groups have shown that both the location and extent of the diaphragmatic defect in nitrofen-induced rat model are comparable to human CDH. Moreover, the rat model shows striking similarities to human CDH regarding PH and cardiovascular defects [25].

Two decades ago studies on nitrofen-induced CDH in rodents led to the dual-hit hypothesis proposing that PH in CDH is the result of two insults [26]. The first insult is represented by an early bilateral nitrofen-induced abnormal development of the pulmonary vasculature [27,28]. The second insult is due to herniation of the abdominal viscera into the thorax, thereby compromising the development of the ipsilateral lung. 

Hampered lung development from intrathoracic herniation of the abdominal viscera has been further studied in surgical animal models; the most commonly used surgical models are lambs and rabbits. In the lamb model, the hernia is surgically created on the 72nd–75th gestational days which correspond to the pseudoglandular stage of lung development. Similarly, the rabbit surgical model is created on the 23rd day of gestation and this model shows some advantages such as shorter gestational period, greater litter size and easier availability [29].

Surgical models are mainly used to investigate interventional strategies in CDH, whereas the nitrofen model has been developed to examine the etiology of CDH highlighting the importance of disturbance in several signaling pathways (retinoid signaling and thyroid signaling) [30,31].

Despite the utility of animal models, the pathogenesis of CDH and the associated PH appears to be largely unsolved and this led to a deeper study of epigenetic modifiers also in animal models. 

miR-200b belongs to the miR-200 family and has a pivotal role in the epithelial-to-mesenchymal transition (EMT) and it is deeply implicated in several organ fibrosis processes via two transcription factors, ZEB1 and ZEB2 [32,33]. The interplay among the miR-200 family and lung pathological entities have been previously studied. 

Yang and colleagues demonstrated that three members of the miR-200 family (miR-200a, miR-200b and miR200c) are downregulated in the lungs of a mouse model of fibrotic lung disease [15].

Fibroblast activation leads to generation of an extracellular matrix (ECM), which is a primary physiological response to tissue injury. The altered balance of ECM synthesis and resolution with excessive ECM production leads to fibrosis and abnormal pulmonary reactivity. ECM production directly depends on three classes of fibroblasts: resident pulmonary fibroblasts, circulating fibroblasts and alveolar epithelial cells (AECs) that undergo a process named EMT. EMT is a complex biological process that encompasses a transcriptional reprogramming of endothelial cells with a shift toward mesenchymal cellular phenotypes. 

The authors found that miR-200 is expressed in the lung AECs at remarkably higher levels than in resident lung fibroblasts and that expression of miR-200 is downregulated in mice with experimental lung fibrosis. These data suggest that downregulation of the miR-200 family in AECs may enhance EMT and contribute to fibroblast accumulation typical of lung fibrosis. [15]

EMT is increasingly recognized as a biological process that contributes to the development of PH; it is a dynamic process controlled by multiple transcriptional factors, such as Zeb1, Zeb2, zinc finger transcription factors Snail (SNAI1), Slug (SNAI2) and basic helix–loop–helix (bHLH) transcription factor Twist-1 [34].

All these transcriptional factors act in concert as repressors and/or activators to drive genetic cell reprogramming and phenotypic switching typical of EMT. 

The role of miR-200 during physiological lung development has been recently investigated in animal models of CDH. Khoshgoo and colleagues generated a miR-200b−/− mouse model to explore the role of miR-200b on lung development, and demonstrated significant alteration of distal airway branching, alveolar wall structure and downregulation of epithelial cell differentiation, with consequent generation of a stiffer or “fibrotic” lung. 

In particular, the authors showed that miR-200b has a key role in maintaining an epithelial cell phenotype during the EMT and experimental data suggest an additional functional role for miR-200 in regulating surfactant protein-B expression and development of surfactant properties. Therefore, the authors demonstrated that miR-200b is required to ensure development of a structurally and functionally effective respiratory organ [16],

Eastwood et al. evaluated miR-200b expression in the lungs of rabbit fetuses with surgically induced diaphragmatic hernia and demonstrated a predominant expression in the terminal bronchi. 

Using in situ hybridization and RT-qPCR, the authors demonstrated that miR-200b-3p levels were significantly higher in the hypoplastic lung when compared to control lungs and tracheal occluded rabbit lungs demonstrated a similar localization of miR-200b when compared with controls [17].

Previous results highlighted the fact that the regulation of miR-200 expression is closely time-dependent and that it may change during the different stages of lung development.

In addition to the miR-200 family, Zhu et al. performed miRNAs microarray analysis of the lungs of nitrofen-induced rats, and they found that miR-33 was significantly decreased in CDH lungs when compared to healthy mouse models [18]. Several genes have been identified as targets of miR-33 and, among these, platelet-derived growth factor alpha-receptors (PDGFRα), serine/threonine kinase PIM-1 and high mobility group AT-hook 2 (HMGA2) were previously found involved in CDH and lung epithelial–mesenchymal interaction [35,36,37].

In addition to the use of miRNAs as diagnostic tools, they have also been studied as a novel prenatal, non-interventional treatment to improve abnormal lung development in the nitrofen-induced rat model of CDH. A recent study demonstrated that miRNAs can also be used as an in vivo therapy. The researchers injected intravenously custom-made in vivo stabilized miRIDIAN miR-200b mimics and observed that transplacental miR-200b therapy corrects abnormal branching morphogenesis in nitrofen-induced lung hypoplasia in pups with no CDH, and reduces the incidence of diaphragmatic defects in the nitrofen rat model of CDH [19].

MiRNA dosing, possible side effects and optimal timing of miR-200b prenatal administration are important ongoing priorities for future studies.

Nevertheless, these animal models have provided new insights into the role of miRNAs in lung hypoplasia and CDH and provide the basis for further investigation on humans.

### 2.2. Human CDH Studies

To date a limited number of studies have begun to explore the role of miRNA in human CDH patients. 

Fabietti and colleagues recently studied EV concentration and associated miRNA expression in amniotic and tracheal fluids of fetuses with CDH undergoing FETO [20]. The authors observed that EV concentration in these fluids was higher in non-survivors than surviving infants following FETO. Moreover, the investigation provided a description of differential miRNA expression in this population. 

Specifically, in the pre-FETO **amniotic fluid** samples of non-survivors there was an over-expression of mir-379-5p and mir-889-3p. Upregulation of mir-379-5p decreases the expression of IGF1, which is a master regulator of Endothelin-1(ET-1), which is implicated in vascular remodeling typical of PH [38,39]. Moreover, miR-889-3p targets fibroblast growth factor receptor 2 (FGFR2) that is critically involved in epithelial cell protection and renewal, and represents a pivotal role in several biological processes including angiogenesis in pulmonary vasculature [40].

In the **tracheal fluid** of post-FETO non-survivors there was an increased expression of mir-223-3p and mir-503-5p, which are both involved in pulmonary smooth muscle cell proliferation and migration [41,42].

One previous study investigated miRNA expression in amniotic and tracheal fluids of fetuses with CDH undergoing the FETO procedure [21]. The authors demonstrated that fetal lungs of CDH between 22 and 25 weeks gestational age show an upregulation of miR200b and miR-10a. Moreover, responders to FETO show an increased expression of the miR-200 family when compared to non-responders. Using semiquantitative in situ hybridization and immunohistochemistry assays, it was demonstrated that miR-200 had an inhibitory effect on TGF-β signaling in human bronchial epithelial cells, whose expression was lower in CDH lungs. The authors concluded that increased miR-200b expression results in decreased target gene expression via decreased TGF-β/SMAD signaling. 

A recent study by Herrera-Rivero et al. explored postnatal miRNA profiles in blood as predictive outcome parameters for CDH patients [22]. At 24 h of life, 7 miRNAs (let-7b-5p, -7c-5p, miR-1307-3p, -185-3p, -8084, -331-3p, -210-3p) were significantly differentially expressed in CDH patients that either died or developed chronic lung disease (CLD). Target gene and pathway analyses indicated that these miRNAs are master regulators of cell cycle regulation, inflammation processes and tissue morphogenesis. 

Although no common target genes for all miRNAs were identified, the authors explored the functional interactions between targets by constructing a protein–protein interaction (PPI) network. They also identified that functional implications were significantly enriched for pathways related to response to growth factors (PI3K-Akt, MAPK, FoxO, Ras, IL4-mediated, TGF-β, integrin, Jak-STAT), ECM-receptor interactions, cell cycle regulation (BTG proteins), regulation of oxygen homeostasis and hypoxia effect (HIF-1-α) and the oxidative stress response. 

Herrera-Rivero et al. suggested that a subset of miRNAs might be used as potential prognostic circulating biomarkers for the development of poor outcomes in CDH newborns. The authors further demonstrated that miRNAs are deeply implicated in TGF-β and semaphorin signaling pathways, as well as inflammatory responses [22].

Postnatal miRNA expression profiles in tracheal aspirates of CDH newborns were recently studied by Piersigilli and colleagues [23]. miR-16, miR-17, miR-18, miR-19b and miR20a were increasingly expressed in CDH patients compared with healthy controls, while miR-19 showed a decreased expression in CDH patients.

All miR-17–92 cluster members (miR-17, miR-17*, miR-18, miR-19a, miR-19b, miR-20 and miR-92a) are subjected to a VEGF-dependent transcriptional control. Through bioinformatic binding prediction tools it has been identified that the miR-17–92 cluster promoter sequence is positively regulated by growth factor signaling via mitogen-activated protein kinase (MAPK). This provides evidence for the role of the VEGF-MAPK-Elk-1 axis on miR-17–92 cluster expression. 

Moreover, deletion of the complete endothelial miR-17–92 cluster in a knockout mouse model determined deleterious effects on vessel morphology, inducing constitutive vessel regression and lowered vascularization [43].

These preliminary human studies support the hypothesis that CDH patients have an altered expression of miRNA profiles, that miRNA signatures might be used to identify high-risk CDH newborns early and that miRNAs and EVs may have therapeutic potential for this condition.

## 3. Discussion and Future Perspective

### 3.1. Understanding the Etiopathogenesis of CDH-Related PH

The etiopathogenesis of CDH is multifactorial and still poorly understood. Single gene mutations account only for few cases, and chromosomal anomalies have been identified only in 10% of cases [44]. The underlying molecular mechanisms remain unknown in the majority of cases [45,46].

Studies on animal models suggested a dual-hit hypothesis, consisting of a primary insult to the lungs before diaphragmatic closure and a second hit to the lungs due to herniation of abdominal organs and mechanical compression of thoracic organs [26]. This model suggests that impaired lung development also occurs independently from the diaphragmatic defect and a deeper insight into the dysregulation of the developmental pathways of lungs is warranted. 

The mortality and disability rates in the long-term follow-ups among CDH patients depend at least partly on severity and long-term persistence of PH. Surviving children often suffer from long-term oxygen dependency, prolonged need for mechanical ventilation and increased risk for pulmonary infections and wheezing [47].

Understanding the causative mechanisms for primary defects of the lung and pulmonary vascular structures observed in CDH is therefore a critical step in global management of this complex condition. 

Aberrant pulmonary vascular development in CDH is postulated to start in early phases of gestation, at 4–16 weeks of gestation. The normal embryogenetic process of lung development starts with initial branching into lung buds and progressive development of terminal bronchioles. Alveolar development starts from the 24th week of gestation and continues throughout the first three years of life. Pulmonary vascular development is parallel to progressive airway branching and is mostly dependent from distal angiogenesis. Distal angiogenesis is characterized by the formation of new capillary endothelium from pre-existing vessels. 

CDH-related PH is highly dependent on pulmonary vascular disruption and pulmonary artery endothelial cell (PAEC) dysfunction [48]. The heterogeneity of the presenting phenotype in CDH patients suggests a complex molecular background resulting from the interplay of genetic, epigenetic and environmental factors. Several molecular pathways have been identified so far as contributors to the pathogenesis of CDH-related PH. Defective retinoid acid signaling [49], nitric oxide (NO) altered responsiveness [50], endothelin-mediated signaling pathway [39] and dysregulated vascular endothelial growth factor (VEGF) expression [51] have been implicated in aberrant pulmonary development, altered angiogenesis and pulmonary vascular response. The lungs are one of the first organs to develop during embryogenesis, and lung development is the result of a complex interplay of transcription factors, morphogens and specific genes. The great variability of lung developmental and function alteration suggests that extragenetic factors regulating gene expression may play a crucial role in the pathogenesis of pulmonary alteration.

### 3.2. Contribution of miRNA in CDH Related PH

Epigenetic modification represents an extremely complex extragenomic mechanism, which regulates gene expression by influencing transcription and translation without involving alterations in the DNA sequence. 

Aberrant DNA methylation, chromatin remodeling, histone modification and microRNA (miRNA) are considered the main means of epigenetic control [52].

In recent years, the focus of epigenetics research has been directed toward miRNAs and their role in pulmonary physiology and pathophysiology. Many miRNAs are expressed in a temporal and spatial manner and are cell- and tissue-specific, having a pivotal role in controlling proliferation, differentiation and apoptosis processes of different cell types. 

Aberrant epigenetic patterns have been suggested as important contributors in the pathogenesis of CDH-related PH in animal models and in vitro human studies. Moreover, altered miRNA expression patterns may impact the development and disease severity of CDH lungs [53]. 

**Figure 1:**
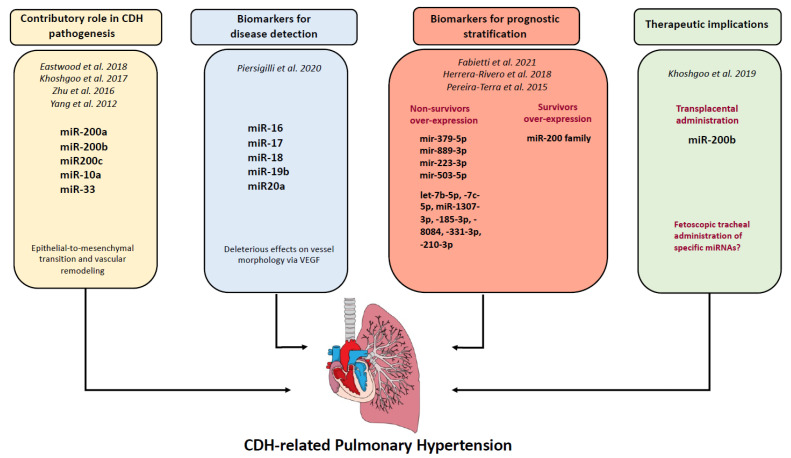
Representative diagram of the multiple roles of miRNAs for CDH-related PH. References [15,16,17,18,19,20,21,22,23] are cited in this figure.

The aim of this comprehensive review was to summarize available data on the role of miRNAs in the pathogenesis of CDH-related PH and to delineate future directions. A summary of our results is shown in Figure 1. Relevant investigations are provided for the reader in Table 1 and Table 2. Considering the limited investigations to date, the exact role of miRNA in lung development and function is still uncertain and sometimes contradictory; this might be attributable to the fact that miRNA-mediated regulation is extremely complex. A broad spectrum of target sites is regulated by each single miRNA, and functional data on miRNA-mediated regulation of gene expression patterns are still limited.

To date, it has been shown that hypoplastic CDH lungs show a specific expression pattern of miR-200 family members [54] with dysregulated miR-200b expression in humans [21] and animal models [17].

miR-200b-deficient mice show macro- and microarchitectural lung changes such as decreased distal airway branching, thicker alveolar walls with reduced airspace and increased number of fibroblast-like cells [16].

miR-200 family members are master regulators of ZEB and TGF-β via the SMAD pathway and regulate pulmonary EMT. Additionally, miR-200b is involved in the regulation of EMT markers (Vimentin) and related transcription factors (Twist) [19], as well as Palate lung and nasal epithelial clone (Plunc), which is essential to the normal airway surface–liquid homeostasis [55].

Moreover, Cadherin-26 (CDH26) is significantly downregulated in the miR-200b−/− lungs, suggesting a role for miR-200b in the regulation of lung epithelial cell differentiation and resultant fibroblast-like phenotype of knockout lungs [16].

Similarly to miR-200, miR-33 plays a regulatory role for EMT [18].

miR-33 targets PDGFRα which is a member of platelet-derived growth factor (PDGF) family and has been previously reported to stimulate smooth muscle cell (SMC) proliferation and over-proliferation of mesenchymal cells with subsequent decreased alveolar airspace [56]. Consistent with previous observations, increased PDGFRα expression has been earlier reported in the lungs of rats with nitrofen-induced CDH [35].

Furthermore, miR-33 also regulates the expression of serine/threonine kinase PIM-1, which is involved in SMC proliferation and resistance to apoptosis, both possible pathogenetic mechanisms implicated in CDH-related PH [57].

HMGA2 is another master target gene of miR-33; HMGA2 activates the Wnt/β-catenin pathway that regulates epithelial cell differentiation of the lung during fetal life. In nitrofen-induced CDH rat lungs, the expression patterns of Wnt7b and Wnt target genes for bone morphogenetic protein 4 (BMP4) are significantly decreased when compared with controls [37].

SMC hyperplasia and increased arterial wall thickness are two of the main aspects of the abnormal lung vascular system found in CDH hypoplastic lungs and evidence regarding miRNAs contribution to these processes is growing. 

Although the complex interplay among miRNAs still has to be clarified, it is reasonable to postulate that miRNAs regulate early lung development by promoting proliferation and EMT. On the other hand, overexpression of specific clusters may lead to severe developmental defects with uncontrolled proliferation and pathological phenotypes of lung epithelial cells resulting in PH.

#### 3.3. miRNAs as Diagnostic and Prognostic Biomarkers

As previously demonstrated, miRNA can also be used as innovative diagnostic tools [21] and biomarkers to evaluate response to therapy [20,21,22]. In recent years, a significant effort has been directed to discovering miRNA biomarkers in order to improve the diagnosis of diseases and early identification of patients with poor prognosis.

As confirmed in the previously cited studies, single miRNAs are not specific for single diseases, while a combination of different miRNAs, referred as miRNA signature, are more likely to reflect the complexity of observed phenotypes. The use of signatures may help to differentiate between patients and improve the overall predictive power of implementing existing disease risk stratification strategies. 

However, single miRNA markers are often the main result of independent studies, while miRNA signatures are less frequently taken into consideration. Future creation of large databases for storing miRNA- signature–disease relation could facilitate the clinical applicability of miRNAs and enhance their clinical value. 

One of the main interesting aspects of miRNAs is that they have been proposed as minimally invasive markers for disease detection. Besides tissues and cell cultures, miRNA patterns can be generated from body fluids (i.e., serum, plasma, blood cells, urine, saliva), which may allow diagnosis and prognosis analysis in a non- or minimally invasive way. 

Although miRNA signatures may represent a valuable hallmark of prognosis, some issues need to be addressed. First, the degree of reproducibility is still under investigation and large validation studies are mandatory. Future profiling of miRNAs in high-throughput settings, with innovative analytical platforms and modern bioinformatic tools is crucial to translate the use of miRNAs from research settings to clinical applications. 

#### 3.4. miRNAs as Therapeutic Targets and Tools

In addition to being valuable candidates as prognostic biomarkers, miRNAs are also appealing therapeutic targets and they also show a possible therapeutic capacity [19,42].

The ongoing morbidity burden related to lung hypoplasia, pulmonary vasculature remodeling and PH warrants the search for better opportunities to normalize prenatal lung development and miRNA administration may be a promising therapeutic option to address abnormal gene expression. 

Although most of the clinically investigated miRNA drugs have been administered via skin or intravenously, miRNA therapeutics can also be provided by inhalation, and this is an encouraging potential route of administration for respiratory diseases to directly target the pathological site whilst minimizing systemic exposure.

To date, inhalation of aerosol miRNA therapeutics has been studied for common respiratory tract conditions as asthma [58] and viral respiratory infections [59], but also for cystic fibrosis [60] and lung cancer [61]. 

miRNA-based drugs have not been studied in human CDH so far but animal models have demonstrated that prenatal transplacental miRNA therapy improves abnormal lung development in cases of CDH [19].

The proportion of side effects of prenatal procedures (i.e., FETO) is still high and this motivates the development for alternative therapies and epigenetic-mediated approaches that may offer a promise of improved survival and reduced morbidity for these patients.

Fetoscopic tracheal administration of specific miRNAs vehiculated in EV may represent an intriguing option to directly target pulmonary hypoplasia and regulate early lung development. 

In addition to PH, CDH-related cardiac dysfunction has been largely recognized as an independent predictor of poor outcomes [62,63,64,65]. Similarly to PH, the molecular background of ventricular dysfunction is largely uninvestigated and epigenetic factors (e.g., miRNAs) may be involved in the pathogenesis. Recent studies have investigated the role of myocardial energetics in CDH animal models, since mitochondrial function has shown to play a key role in heart failure [66]. A recent study demonstrated a significant downregulation of mitochondrial-biogenesis-related genes in CDH tissue in the nitrofen-induced CDH rat model [67]. These preliminary findings stimulate further investigation on the role of epigenetic modulators also in the context of CDH-related cardiac dysfunction. 

However, there are still practical issues to solve to facilitate the future therapeutic use of miRNAs, including the identification of optimal administration routes, the control of pharmacokinetics, the targeting of specific tissues, and the study of intracellular effects. However, the field of miRNA research in CDH seems undoubtedly appealing. 

The role of epigenetics in the development of PH is a rapidly growing field of research.

A better understanding of the pleiotropic effects of miRNAs in several regulatory networks is a major challenge for the management of CDH-related PH to identify diagnostic markers, prognosis determinants and apply novel treatment strategies for targeting epigenetic dysregulation.

Early recognition of miRNAs expression profiles may help in the stratification process to identify high-risk CDH neonates; however, a detailed and comprehensive characterization of miRNAs in larger cohorts is necessary for both their potential diagnostic and therapeutic use.

## Figures and Tables

**Table 1:** Summary of current investigations on miRNA in CDH animal models and pulmonary hypertension and hypoplasia.

Study, Year	Material	MiRNAs	Results	Possible Effect/Affected Pathways
Animal Models				
Yang et al., 2012 [15]	Nitrofen rat model	miR-200a; miR-200b; miR200c	Downregulated	Fibrotic lung disease
Khoshgoo et al., 2017 [16]	Nitrofen rat model	miR-200b	miR-200b−/− knockout model	Altered airway branching, alveolar wall structure and downregulation of epithelial cell differentiation, fibrotic lung
Eastwood et al., 2018 [17]	Rabbit model	miR-200b-3p	Overexpressed	Overexpression in hypoplastic lung
Zhu et al., 2016 [18]	Nitrofen rat model	miR-33	Decreased expression	Decreased expression in CDH lungs, altered lung epithelial–mesenchymal interaction
Khoshgoo et al., 2019 [19]	Nitrofen rat model	miR-200b	Compensatory upregulation of miR-200b in less hypoplastic lungs	Transplacental miR-200b therapy corrects abnormal branching morphogenesis

**Table 2:** Summary of current investigations on miRNA in CDH human studies.

Study, Year	Material	MiRNAs	Results	Possible Effect/Affected Pathways
Human studies				
Fabietti et al., 2021 [20]	Amniotic and tracheal fluids of fetuses with CDH undergoing FETO	mir-379-5p; mir-889-3p	Overexpression in pre-FETO amniotic fluid samples of non-survivors	Vascular remodeling, epithelial cell protection and renewal
Fabietti et al., 2021 [20]	Amniotic and tracheal fluids of fetuses with CDH undergoing FETO	mir-223-3p; mir-503-5p	Overexpression in tracheal fluid of post-FETO non-survivors	Pulmonary smooth muscle cell proliferation and migration
Pereira-Terra et al., 2015 [21]	Amniotic and tracheal fluids of fetuses with CDH undergoing FETO	miR200b; miR-10a	Upregulation in fetal lungs of CDH. Increased expression of miR-200 in FETO responders	Decreased TGF-β/SMAD signaling in human bronchial epithelial cells
Herrera-Rivero et al., 2018 [22]	Blood of CDH newborns, collected 24 h after birth	let-7b-5p, -7c-5p, miR-1307-3p, -185-3p, -8084, -331-3p, -210-3p	Differentially expressed in CDH non survivor or developing chronic lung disease	Cell cycle regulation, inflammation processes and tissue morphogenesis
Piersigilli et al., 2020 [23]	Tracheal aspirates of CDH newborn	miR-16, miR-17, miR-18, miR-19b, miR20a	Differentially expressed in CDH patients compared with healthy controls	(TGF)-β signaling pathway, early lung branching, morphogenesis and alveologenesis

## Data Availability

Not applicable.
